# Mistakes from the HIV pandemic should inform the COVID-19 response for maternal and newborn care

**DOI:** 10.1186/s13006-020-00306-8

**Published:** 2020-07-25

**Authors:** Karleen Gribble, Roger Mathisen, Mija-tesse Ververs, Anna Coutsoudis

**Affiliations:** 1 School of Nursing and Midwifery, Western Sydney University, Locked Bag 1797, Penrith, NSW 2751 Australia; 2 Alive and Thrive Southeast Asia, FHI 360, 60 Ly Thai To Street, Hanoi, Vietnam; 3 Center for Humanitarian Health, Johns Hopkins Bloomberg School of Public Health, Baltimore, MD 21205 USA; 4 Department of Pediatrics and Child Health, School of Clinical Medicine, University of KwaZulu-Natal, Durban, South Africa

**Keywords:** COVID-19, HIV, Prevention of mother-to-child-transmission, Infant and young child feeding in emergencies, Policy development

## Abstract

**Background:**

In an effort to prevent infants being infected with SARS-CoV-2, some governments, professional organisations, and health facilities are instituting policies that isolate newborns from their mothers and otherwise prevent or impede breastfeeding.

**Weighing of risks is necessary in policy development:**

Such policies are risky as was shown in the early response to the HIV pandemic where efforts to prevent mother to child transmission by replacing breastfeeding with infant formula feeding ultimately resulted in more infant deaths. In the COVID-19 pandemic, the risk of maternal SARS-CoV-2 transmission needs to be weighed against the protection skin-to-skin contact, maternal proximity, and breastfeeding affords infants.

**Conclusion:**

Policy makers and practitioners need to learn from the mistakes of the HIV pandemic and not undermine breastfeeding in the COVID-19 pandemic. It is clear that in order to maximise infant health and wellbeing, COVID-19 policies should support skin-to-skin contact, maternal proximity, and breastfeeding.

## Background

The care of mothers and infants in the COVID-19 pandemic has proven challenging, as policy makers have grappled with how to protect newborns when their mothers are suspected or confirmed as having COVID-19. For the general population, isolation of the infected from the uninfected and physical distancing are essential to preventing disease transmission and achieving good health outcomes. However, mothers and infants present a special situation as the risk of mother-to-child transmission of SARS-CoV-2 needs to be weighed against the protection from infectious diseases and the support for bonding and caregiving provided by close maternal proximity and breastfeeding.

In low-, middle- and high-income countries, some policy makers and practitioners appear to have given more weight to the risk of SARS-CoV-2 transmission than the consequences of maternal separation and reducing breastfeeding. At the most extreme, infants are being isolated from their mothers with suspected or confirmed COVID-19 for periods of up to 2 weeks (e.g. [1, 2]). In some contexts, expressed breastmilk may be provided, while in others even provision of breastmilk is prohibited. More commonly, skin-to-skin contact after birth is being withheld and where infants are permitted to share a room with their mothers, a distance of two meters or a screen separates them (e.g. [3, 4]). These decisions reduce infant access to breastfeeding and breastmilk and have been made on the basis of very little evidence. A similar weighing against breastfeeding was made in the early response to the HIV pandemic.

## Discussion

### Experience of the HIV pandemic

The possibility of mother-to-child transmission of HIV through breastfeeding was first raised in 1985 in a letter published in the Lancet [5]. In this case, an Australian woman who had been given an HIV contaminated blood transfusion for a postpartum haemorrhage, contracted HIV and her infant was also found to be HIV positive [5]. It was proposed that the mother transmitted HIV to her infant through breastfeeding. Shortly thereafter, HIV was isolated in breastmilk [6]. The fear of mother-to-child transmission of HIV through breastmilk led authorities in the USA to quickly respond by recommending that HIV-positive mothers should not breastfeed [7]. Policy makers in the developing world followed suit and HIV-positive women were supplied with free infant formula despite there being no knowledge of the magnitude of risk of HIV transmission through breastfeeding [8].

Replacement infant formula feeding by HIV-positive women was quickly institutionalised in many countries. However, it was not until 1992 that a reliable estimate of HIV transmission through breastmilk was published indicating that after 24 months of breastfeeding 14% of breastfed infants risked contracting HIV [9]. Further research highlighted that this was the cumulative risk and not, as supposed, a one-off risk when mothers commenced breastfeeding, and breastfeeding for a shorter period of 6 months carried a risk of about 4% [10]. In addition, if breastfeeding during the first 6 months was exclusive, this risk was even further diminished. It emerged that supporting exclusive breastfeeding maximized infant HIV-free survival [11].

Rather than improving infant health, policies to move away from supporting breastfeeding in the HIV pandemic had a devastating impact on infant mortality in many middle- and low-income countries. More infants lost their lives through diarrhoea and pneumonia related to infant formula feeding compared to those who lost their lives through HIV infection [11]. The hard lesson was learnt that an HIV negative dead infant is still dead. The aftermath of these recommendations had serious repercussions that lasted for more than a decade as the fear of HIV transmission and the normalisation of bottle feeding changed infant feeding practices. Even in communities that previously had a strong breastfeeding culture, mothers who were HIV-negative opted to infant formula feed with attendant increases in infant morbidity and mortality [11].

### Differences between HIV and SARS-CoV-2 infection in infants

While policy development in the HIV pandemic might share some similarities with the COVID-19 pandemic, the method of transmission and the impact of infection on infants are quite different. HIV is a blood borne infection that can be transmitted in utero, during birth, and via breastmilk [12]. HIV can cause serious disease, and before the introduction of effective anti-retroviral treatment, mortality rates of those infected with HIV as infants approached half by 3 years of age [13].

In contrast, SARS-CoV-2 appears to be primarily transmitted through respiratory droplets and contact routes [14]. To date there is no clear evidence that vertical transmission occurs and samples of amniotic fluid, and cord blood taken from mothers with confirmed COVID-19 have been found negative for SARS-CoV-2 [15]. In a small number of cases, viral RNA particles have been detected in expressed breastmilk, but no live virus has been found and breastmilk is not thought to be a transmission route [16, 17]. Antibodies to SARS-CoV-2 have been found in breastmilk suggesting that breastfeeding may provide specific protection against the virus [18]. A recent review of infants born to 655 women with COVID-19 concluded that it was rare for infants to be infected with SARS-CoV-2 and that rates of infection are not increased when infants remain proximate to their mothers and breastfeed [17]. Children infected with SARS-CoV-2 are mostly asymptomatic or have mild symptoms [19]. It seems that fever and cough are relatively the most reported symptoms amongst paediatric cases and many children with SARS-CoV-2 infection might remain undetected. Complications and death appear to be rare [19].

As described, HIV and COVID-19 are starkly different in terms of transmission method and consequences of transmission. Nonetheless, it should be noted that despite the possibility of HIV being transmitted via breastmilk and that, prior to antiretroviral use, infection with HIV was commonly fatal, policies to move away from breastfeeding in the HIV pandemic caused great harm. Since there is no evidence that SARS-CoV-2 is transmitted via breastmilk and COVID-19 is rarely serious in infants, the relative harm of policies impeding breastfeeding in the COVID-19 pandemic may therefore be even greater than was the case for the HIV pandemic.

### World Health Organization guidance

**Fig. 1:**
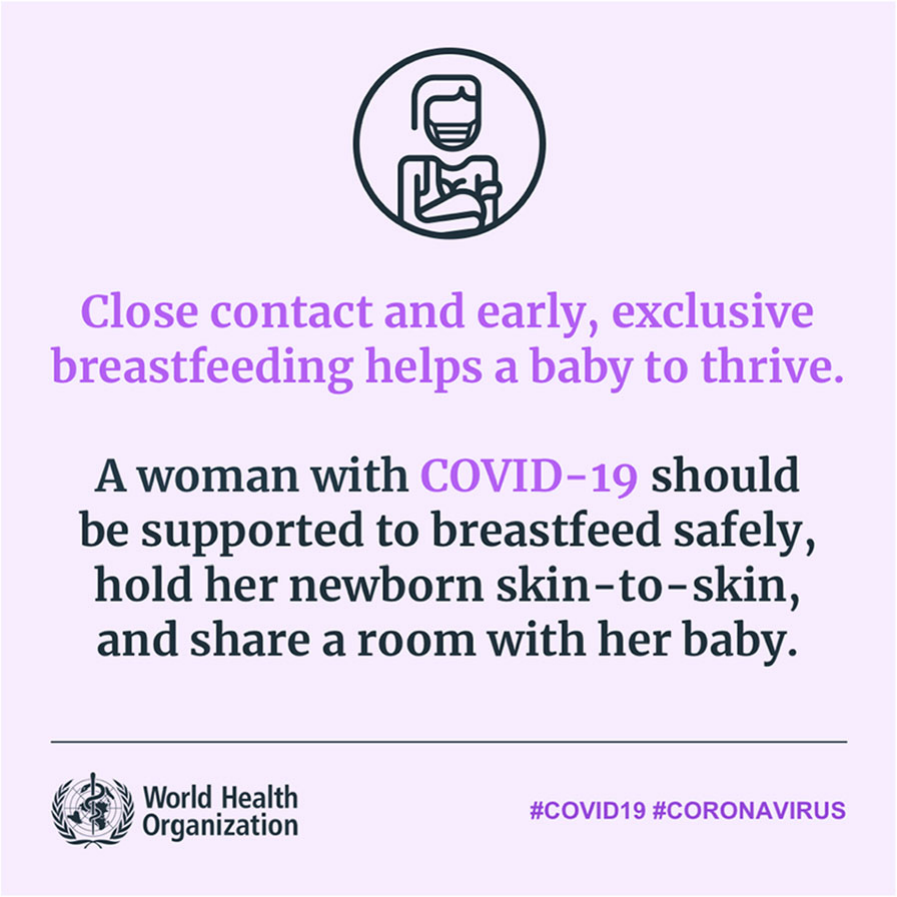
WHO infographic supporting maternal proximity, skin-to-skin contact, and breastfeeding for women who have COVID-19

**Fig. 2:**
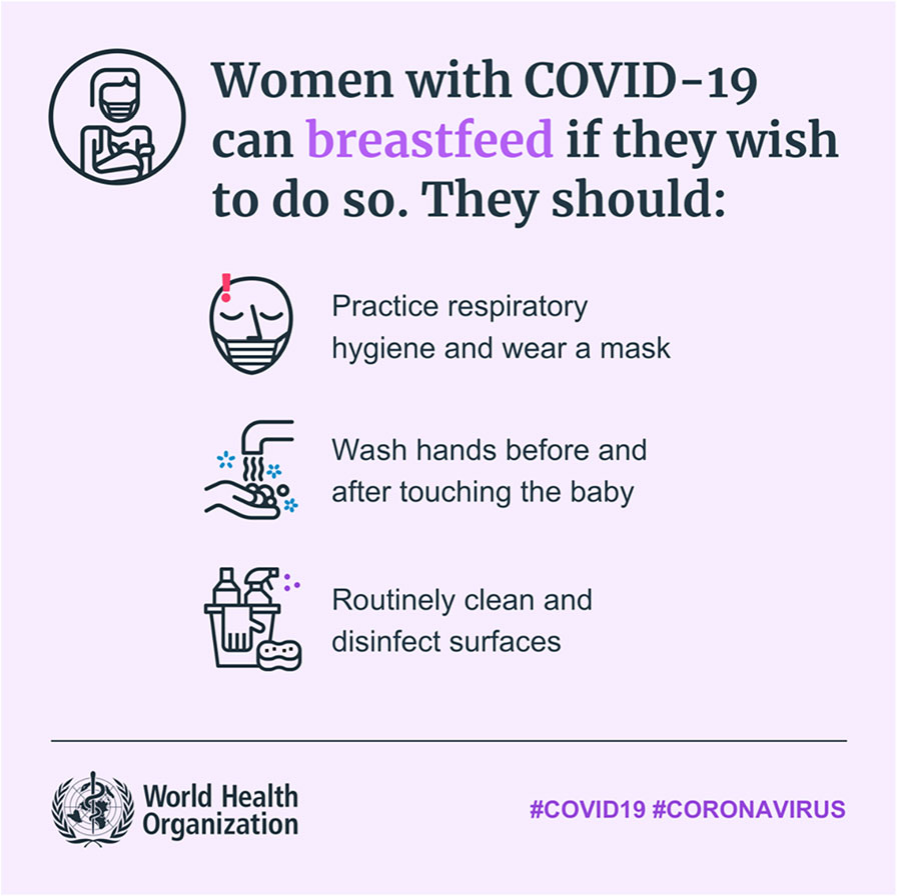
WHO infographic illustrating the hygiene measures to be undertaken by breastfeeding women who have COVID-19

**Fig. 3:**
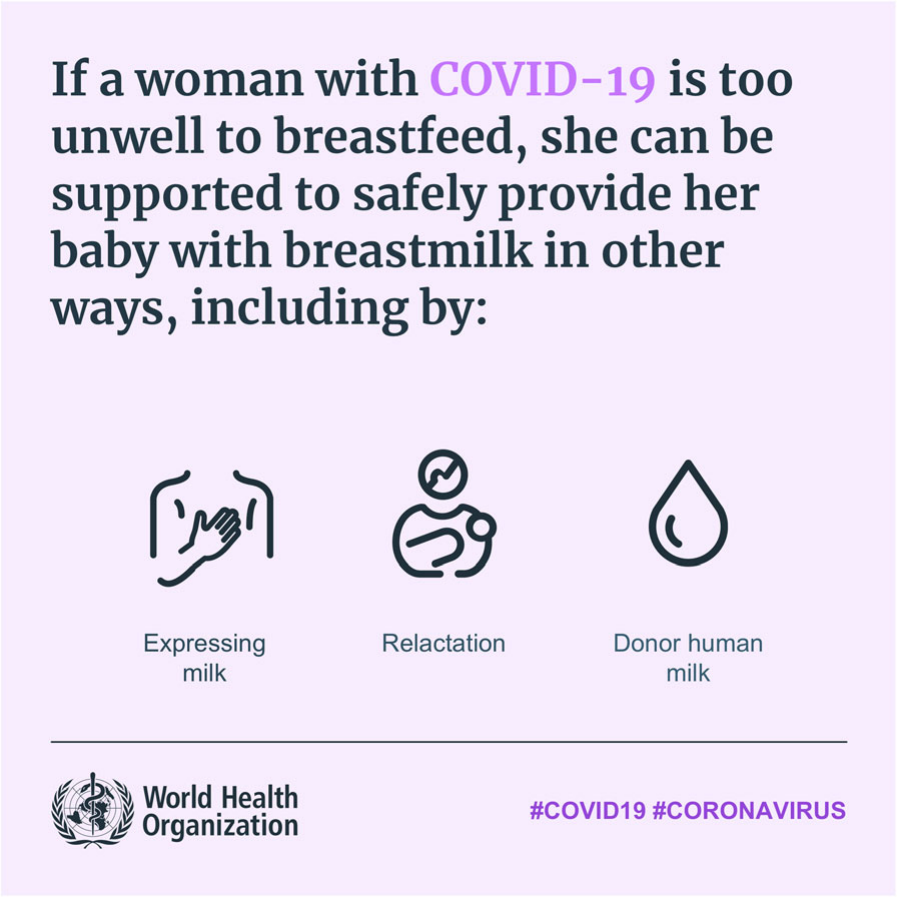
WHO infographic presenting breastmilk options if mothers are too ill with COVID-19 to breastfeed

On 13 March 2020 the WHO published interim guidance on the clinical management of COVID-19, including on the care of newborn infants [20]. This guidance was heavily weighted towards supporting maternal proximity and breastfeeding. It states that women with suspected, probable or confirmed COVID-19 should be supported to have their infants placed skin-to-skin with them immediately after birth, initiate breastfeeding within an hour of birth, to keep their infants by their side day and night, and exclusively breastfeed [20]. Alongside these practices, mothers should apply infection prevention and control measures by practicing respiratory hygiene, washing their hands before and after contact with their infants, and ensuring that surfaces that they have been in contact with are cleaned and disinfected [20]. Where mothers are unable to breastfeed because of illness they are to be assisted to express milk for their infants [20]. If this is not possible, the use of donor human milk should be explored and if this is not available wet nursing or infant formula may be considered. Ongoing milk expression and relactation when mothers are well enough is also recommended [20]. Infographics to assist in the promotion of the guidance were released by the WHO, as shown in Figs. 1, 2 and 3. The WHO released updated guidance on the management of COVID-19 on 27 May 2020, the recommendations regarding maternal and newborn care were unchanged [21]. The rationale for these recommendations is based on the importance of breastfeeding in preventing infant morbidity and mortality [20–22].

### Importance of breastfeeding

Worldwide, 72% of hospital admissions for diarrhoea and 57% of admissions for respiratory infections can be attributed to a lack of breastfeeding [23]. In low- and middle- income countries, infants who are not breastfed have a mortality rate that is eight times greater than exclusively breastfed infants and globally more than 800,000 lives could be saved if breastfeeding were universally practiced [23]. Even in high-income countries, hospitalisation rates are greatly elevated in non-breastfed infants [24]. The WHO COVID-19 guidance, notes that health service practices should minimise disruption to breastfeeding [21]. However, COVID-19 policies that require isolation of mothers and infants and denial of breastmilk prevent infants from having access to breastfeeding. Withholding skin-to-skin contact after birth reduces rates of exclusive breastfeeding by one third and impedes maternal bonding and sensitivity [25, 26]. Any increase in physical distance between mothers and infants reduces the frequency of breastfeeding and may therefore negatively impact on the establishment of breastfeeding [27]. Reduction of breastfeeding not only has an impact on infant physical health, but adversely impacts infant cognitive development [28]. It can also reduce maternal caregiving capacity and so result in increased rates of child maltreatment [29]. Maternal deaths due to breast and ovarian cancers as well as type 2 diabetes can also be attributed to short breastfeeding duration practices [28].

### Exploitation of the pandemic by the infant formula industry

Policies that impede breastfeeding also enhance the marketing of infant formula as manufacturers can argue that normal prohibition on donations of infant formula do not apply because mothers cannot breastfeed. Donations are a powerful marketing tool that are proscribed by the WHO International Code of Marketing of Breastmilk Substitutes and the Operational Guidance on Infant and Young Child Feeding in Emergencies [30, 31]. However, in the COVID-19 pandemic, infant formula manufacturers and distributors are donating to hospitals, governmental and non-governmental agencies, and individuals worldwide [32–34]. The negative short and long-term harms of donations in emergencies are well known [35]. At all times, but especially in emergencies, protection and support for breastfeeding needs to be strengthened and not undermined [36].

## Conclusion

COVID-19 policy makers must learn from past mistakes. In 2010 this journal published a thematic issue on *“HIV and Infant Feeding: Lessons Learnt and Ways Ahead”* in which Moland et al. [37] concluded, *“The scientific evidence base warns against hasty dismissal of the evolved benefits of breastfeeding … In future, the global health professional community should be more sceptical of claims about the risks of breastfeeding*.” We stand now in the future spoken of, facing a new pandemic. With such substantial, well documented evidence of the importance of maternal proximity and breastfeeding for child survival, development, and health as well as the protection that breastfeeding affords mothers, we cannot repeat the mistakes of the HIV pandemic. Breastfeeding should not be interrupted because of a fear that SARS-CoV-2 could be transmitted from mothers to infants during breastfeeding and so harm infants. Countries, professional associations, and health facilities should follow the lead of the WHO, taking heed of the experiences of the past and take care in the COVID-19 response to not undermine breastfeeding.

## Data Availability

Not applicable.
